# Immune cell population dynamics following neonatal BCG vaccination and aerosol BCG revaccination in rhesus macaques

**DOI:** 10.1038/s41598-024-67861-6

**Published:** 2024-07-23

**Authors:** Laura Sibley, Charlotte Sarfas, Alexandra L. Morrison, Jessica Williams, Konstantinos Gkolfinos, Adam Mabbutt, William Eckworth, Steve Lawrence, Mike Dennis, Andrew White, Sally Sharpe

**Affiliations:** UK Health Security Agency, Porton Down, Salisbury, SP4 0JG UK

**Keywords:** Immunology, Vaccines

## Abstract

The BCG vaccine is given to millions of children globally but efficacy wanes over time and differences in the immune systems between infants and adults can influence vaccine efficacy. To this end, 34 rhesus macaques were vaccinated with BCG within seven days of birth and blood samples were collected over 88 weeks for quantification of blood cell populations. Overall, the composition of cell populations did not change significantly between BCG vaccinated and unvaccinated groups, and that BCG vaccination did not perturb normal development. In comparison to adult macaques, higher numbers of CD4+ T-cells, Tregs and NK cells were measured in the infant age group, suggesting a potential bias towards immunosuppressive and innate immune populations. Antigen-specific IFNγ secreting cell frequencies in infant BCG vaccinated animals were detectable in peripheral blood samples for 36 weeks after vaccination but declined following this. To evaluate the long-term impact of infant BCG vaccination on subsequent revaccination with BCG, a pilot study of three adult macaques received an aerosol BCG revaccination approximately 3 years after their initial BCG vaccination as infants. This induced an increase in PPD-specific IFNγ secreting cells, and increased secretion of the cytokines IFNγ and IL-1β, following stimulation with other microorganisms, which are signals associated with trained innate immunity.

## Introduction

Tuberculosis (TB), a disease caused by infection with *Mycobacterium tuberculosis*, accounted for 1.6 million deaths and 10.6 million cases in 2022 worldwide^1^. The vaccine; Bacille Calmette-Guerin (BCG) is the only licenced vaccine for TB and is given at birth in many countries as part of routine childhood vaccination campaigns^2^. The BCG vaccine has been shown to provide protection against childhood TB, particularly TB meningitis and disseminated disease^3^. BCG delivered at birth has also been shown to protect against other unrelated diseases and reduces infant mortality caused by non-tuberculous diseases by up to 40%^4,5^.

Due to the vast numbers of TB cases, the variable efficacy of BCG and the waning of its effectiveness over time, there is a drive for novel replacement vaccines, or for new, more efficacious, vaccination strategies that incorporate BCG to reduce the global burden of TB so that it is no longer a public health challenge by 2030, as set out in the WHO StopTB plan^6^. Due to the excellent safety profile, cost-effectiveness and established worldwide distribution chain for the BCG vaccine, it is likely that BCG will remain in use for some time to come. Despite BCG failing to induce long lasting immunity against TB, there is evidence that BCG can have long lasting impacts on the immune system, including inducing trained innate immunity and off-target protection. Given the substantial number of people vaccinated with BCG at birth, it is important to understand the impact of infant BCG vaccination later in life. This is especially imperative given the invent of vaccines aiming to boost the BCG immune response in adolescence or adulthood for the purposes of enhancing waning TB protection. An understanding of the potential influence on the immune response to novel vaccines or booster vaccinations, and in particular whether is evidence of masking or blocking of these responses, will influence vaccination strategies going forward.

The infant immune system is different to that of adults in terms of maturity and a more limited exposure to environmental stimuli. An infant’s internal microbiome, which shapes and influences the immune system and how it responds to future infections, may not have fully developed^7,8^. Furthermore, there is thought to be a bias towards Th2 profile immune responses in infants to prevent inappropriate inflammatory immune responses to maternally derived antibodies^9^, which may also alter the immune response induced by vaccinations delivered in infancy. Previous investigations of infant BCG vaccination in humans have shown that, in comparison to unvaccinated infants, infants possess more effector memory T-cells three months after vaccination, and a lower percentage of late differentiated T-cells and apoptotic T-cells at 13 months, but there is limited overall impact on proportions or absolute cell counts^10^. Mycobacteria-specific polyfunctional T-cells, secreting interferon gamma (IFNγ), tumour necrosis factor alpha (TNFα) and interleukin 2 (IL-2), are induced following BCG vaccination of infants^11^, but it has been demonstrated that infant T-cells produce less, and a different array of, cytokines following mycobacterial antigen stimulation than an adult’s^12,13^. These age-related differences indicate that the use of age-matched animal models in vaccine evaluation experiments, may provide a better understanding of the vaccine’s effects in particular target populations^14^.

Non-human primates are the most clinically relevant animal model, and closely resemble human physiology, immunology and develop similar patterns of TB disease to humans^15,16^. In order to further characterise the infant immune system and immune response following BCG vaccination, a cohort of infant rhesus macaques was vaccinated within 7 days of birth to assess changes in the phenotype and function of peripheral blood cell populations using a haematology analyser, flow cytometric whole blood immunophenotyping, and an ex-vivo IFNγ ELISPOT to measure the frequency of mycobacteria-specific IFNγ secreting cells over the course of 88 weeks.

Revaccination with BCG is one of the strategies for controlling TB infections that is under evaluation in clinical trials^17,18^. While revaccination using intradermal injection is generally the route of vaccination under evaluation in human clinical trials, preclinical studies have shown BCG to be more efficacious when delivered by the respiratory^19–21^ or intratracheal route^22^. Delivery of BCG to the same site where airborne M.tb causes infection should activate cells at the portal of pathogen entry and induce quicker and more relevant immune responses that systemically delivered BCG. The aerosol route has the additional advantage of being needle-free and portable handheld nebuliser devices which are small, cheap, and easy to use, make it a viable approach for vaccine delivery in resource limited settings. To investigate the potential offered by revaccination targeted to the lung, a pilot study was conducted with a group of three macaques that received BCG at birth and a booster aerosol BCG revaccination at approximately 3 years old. Cell population profiles were monitored using a haematology analyser, and functional immune responses characterised using an IFNγ ELISPOT assay. As BCG vaccination is thought to impart long term epigenetic changes on the infant immune response, which may confer protection against unrelated diseases, the induction of trained immunity was assessed through the measurement of secreted cytokines following stimulation with whole cell inactivated microorganisms, unrelated to TB. Applying these methods to samples collected from the cohort of infants vaccinated with BCG at birth, we investigated whether long-term changes were apparent in the immune response to non-TB organisms, and whether aerosol BCG revaccination alters the trained immune response.

## Results

### Rhesus macaques exhibit increased CD4+ and Treg cell compartments in the first year of life

The concentrations of lymphocyte (CD4, CD8, regulatory T cells (T regs), Natural killer T cells (NK), gamma-delta T cells (γδ) and monocyte (CD14+ CD16+, CD14+ CD16−, CD14− CD16+) subpopulations were defined in blood samples collected from three groups of naïve, unvaccinated macaques of different ages using immunostaining and flow cytometry. The data to enable comparison across the age groups was obtained from two cohorts, the first cohort of nine macaques was sampled at either of 6 or 12 weeks of age and at year one. A second cohort of 12 animals aged between 3 and 4 years’ old was used to provide profiles to enable comparison between age groups. ‘Year < 1’ represents a combination data collected at either of 6 or 12 weeks of age, as samples were only collected at one timepoint and is referred to as ‘year zero’.

**Figure 1:**
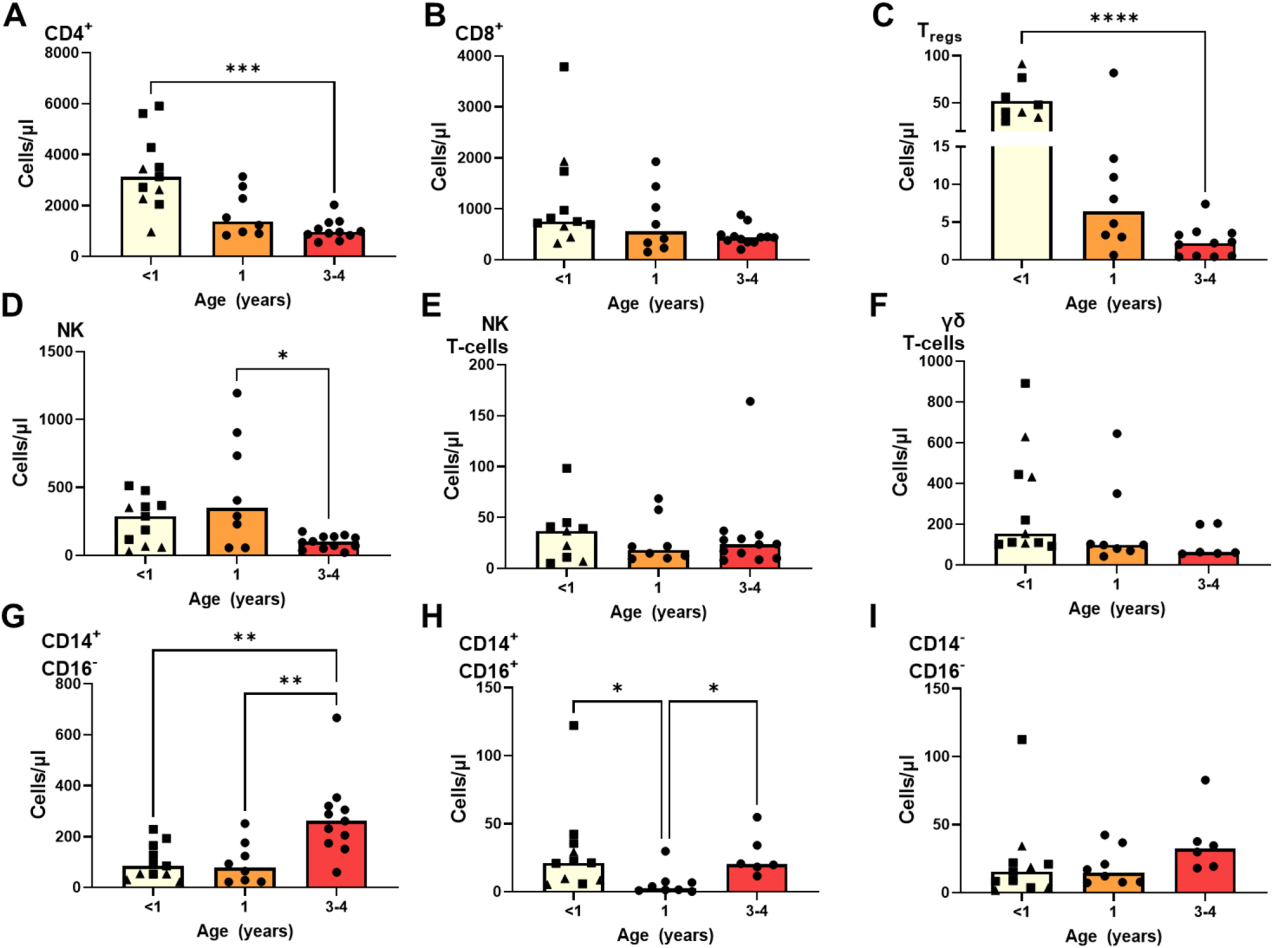
Cell populations measured in unvaccinated rhesus macaques aged < one, one and 3–4 years using whole blood immunophenotyping. (A) CD4+ T-cells, (**B**) CD8+ T-cells, (**C**) Tregs, (**D**) NK cells, (**E**) NK T-cells, (**F**) γδ T-cells, (**G**) CD14+ CD16− monocytes, (H) CD14+ CD16+ monocytes, (**I**) CD14− CD16+ monocytes. Bars show group medians with individual values indicated by dots. Asterisks indicate Mann–Whitney test comparisons carried out between groups *p   ≤ 0.05, **p   ≤ 0.01, ***p   ≤ 0.001; Triangles indicate data sampled at 6 weeks of age, squares indicate data sampled at 12 weeks of age**.**

The numbers of CD4+ T-cells present in peripheral blood were significantly higher in the first few weeks of life (year 0) in comparison to the levels determined at years 1 and 3 (*p* = 0.0008) (Fig. 1A), in contrast the numbers of CD8 + T-cells were found to be similar across the age groups (Fig. 1B). The number of regulatory T cells significantly declined between year 0 and year 3 (*p* = *0.0047*) (Fig. 1C) as did the number of NK cells (*p* = 0.0317) (Fig. 1D), although numbers did remain stable between year 0 and year 1. NK T-cells and γδ T-cell numbers did not significantly change between the time points analysed (Fig. 1E, F). There was a significant increase in the quantity of CD14+ CD16− monocytes in macaques aged between 3 and 4 years in comparison to that determined in infants aged less than 1 year (*p* = 0.0068) and those of 1 year of age (*p* = 0.0098) (Fig. 1G). In contrast the number of CD14+ CD16+ monocytes were found to be significantly lower at year 1 of age in comparison to the levels measured at both year 0 (*p* = 0.0193) and year 3 (*p* = 0.0356) (Fig. 1H). Whilst levels of CD14− CD16+ monocytes did not change significantly, a non-significant trend for increased counts of this cell population to be measured at year 3 (Fig. 1I) was seen.

### Peripheral blood cell population dynamics during the first 18 months of life in BCG vaccinated and unvaccinated rhesus macaques

**Figure 2:**
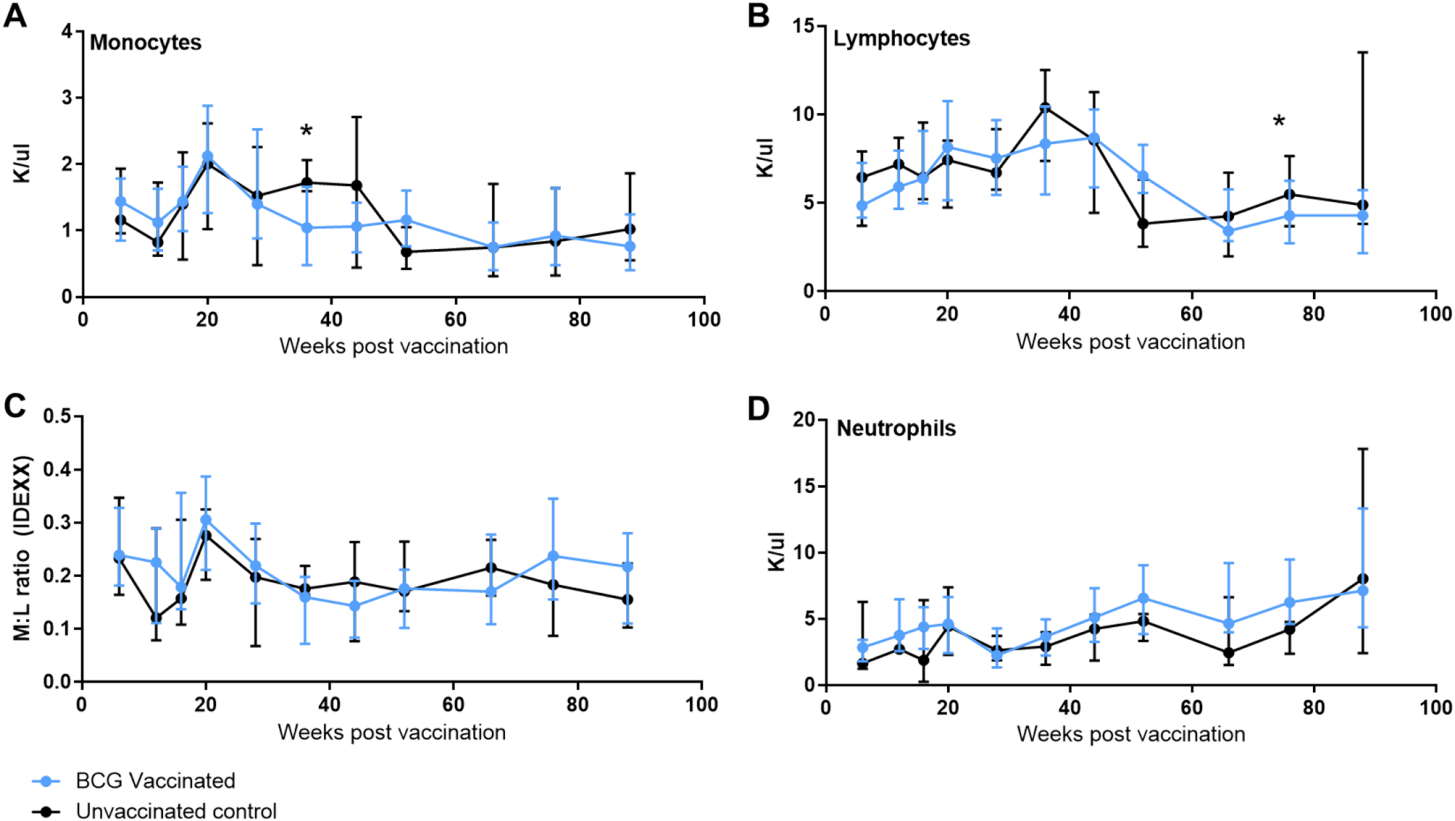
Cell populations measured in whole blood using the IDEXX ProCyte haematology analyser following BCG vaccination within seven days of birth during the first 18 months of life. (**A**) Monocytes, (**B**) lymphocytes, (**C**) M:L ratio, (**D**) neutrophils. Measured in K (1000 cells)/μl. Medians shown with interquartile range shown. Mann–Whitney U-test used to compare between groups *p   ≤ 0.05. Blue: BCG vaccinated (n = 34), black: unvaccinated (n = 9).

The number of lymphocytes, monocytes and neutrophils per μl of peripheral blood was measured six weeks after BCG vaccination, and then at regular intervals until week 88 using an IDEXX ProCyte haematology analyser. Monocyte numbers showed similar general trends in both vaccinated and unvaccinated animals until week 36 when significantly lower monocyte counts were observed in the vaccinated group (*p* = 0.0309) (Fig. 2A). In contrast numbers remained stable in the unvaccinated group until week 52 when decreased counts were also observed in this group. Subsequently, numbers in both the vaccinated and unvaccinated groups plateaued. In both groups, lymphocyte numbers increased from weeks 6 to 44, and then declined between weeks 44 and 66 (Fig. 2B). Seventy-eight weeks after vaccination, significantly fewer lymphocytes were present in the vaccinated group than in the unvaccinated group (*p* = 0.0083). Similar trends were observed in the monocyte:lymphocyte (M:L) ratio between the vaccinated and unvaccinated groups (Fig. 2C), with M:L ratios decreasing between weeks six and 12, then increasing at week 20 after which ratios stabilised until the end of the study period. The kinetics of neutrophil numbers were also similar between the groups with both vaccinated and unvaccinated animals showing a trend for numbers to increase steadily over the 88-week study period (Fig. 2D).

### Lymphocytes and monocyte populations change temporally throughout the first 18 months of life

**Figure 3:**
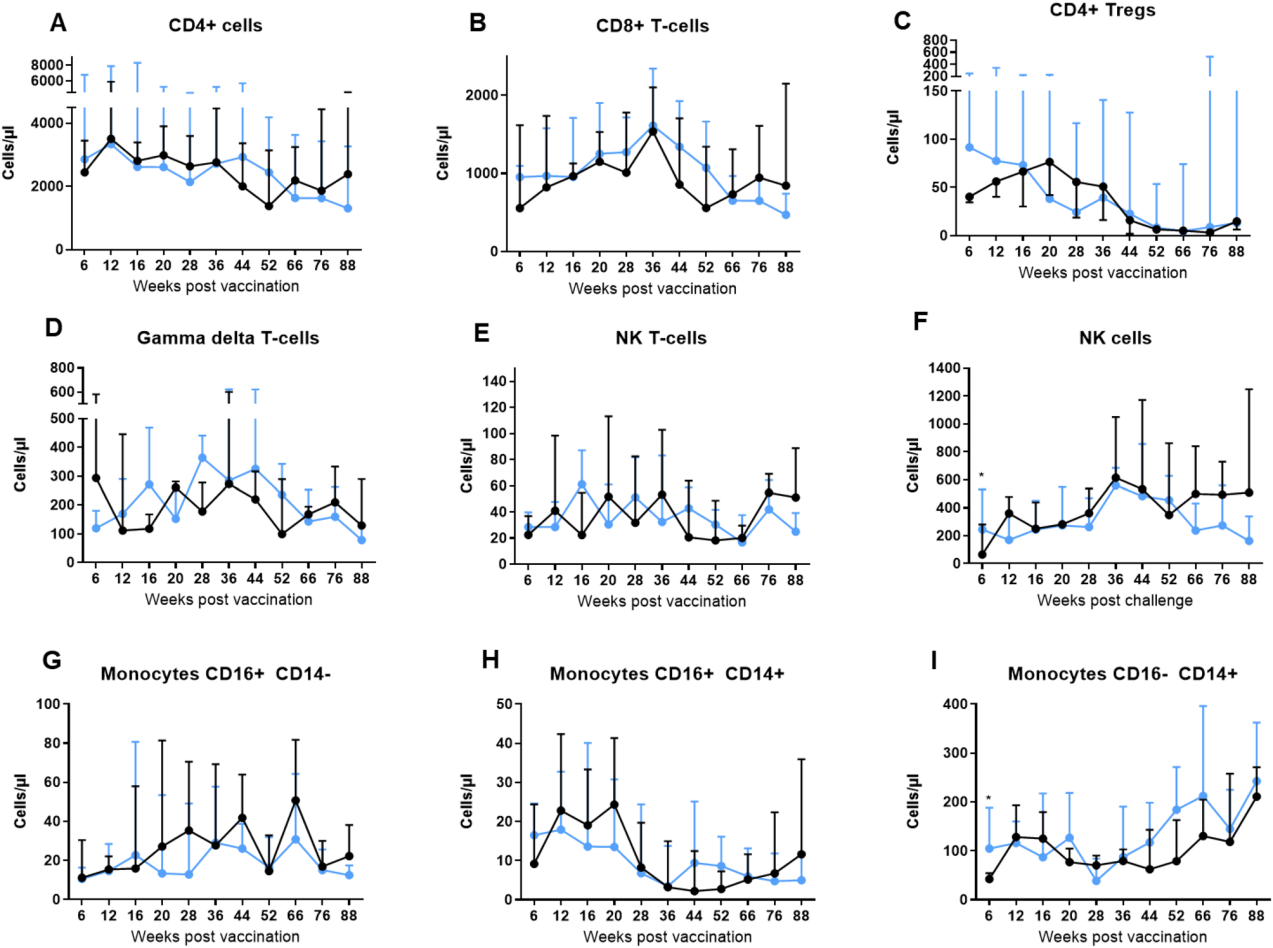
Cell populations measured in whole blood using immunophenotyping following BCG vaccination within 7 days of birth. (**A**) CD4+ T-cells, (**B**) CD8+ T-cells, (**C**) CD4+ Tregs, (**D**) Gamma delta T-cells, (**E**) NK T-cells, (**F**) NK cells, (**G**) CD14+ CD16− monocytes, (**H**) CD14+ CD16+ monocytes, (**I**) CD14− CD16+ monocytes. Cells measured as absolute counts/ml of blood. Blue: BCG vaccinated (n = 34), black: unvaccinated (n = 9). Medians shown with interquartile range. Mann–Whitney-U tests between groups carried out,*p  ≤ 0.05.

The levels of lymphocyte and monocyte subpopulations were evaluated in blood samples collected at regular intervals throughout the study period by immunostaining and flow cytometry. CD4+ T-cell numbers were very similar between the vaccinated and unvaccinated groups and declined over time, but did not change significantly (Fig. 3A). In contrast, CD8+ T-cells showed a different trajectory to that of the CD4+ T-cells (Fig. 3B) with a trend for the BCG group to possess higher numbers of CD8+ T cells 6 weeks after vaccination, and numbers in both groups increased steadily to a peak at week 36, whereupon numbers started to decrease. This decline in CD8+ T cell numbers continued until the end of study at week 88 in the BCG vaccinated group, whereas numbers in the unvaccinated group increased once more at week 66, although differences between CD8+ T-cell numbers in vaccinated and unvaccinated groups did not reach significance at this time point (*p* = 0.1039).

An initial non-significant trend for BCG vaccinated macaques to possess more CD4+ Tregs at weeks 6 and 12 (*p* = 0.1167 and *p* = 0.5899 respectively) (Fig. 3C) than the unvaccinated group was observed, although numbers measured in the unvaccinated animals progressively increased reaching similar levels to those in the BCG group by week 16. Regulatory CD4+ T cell numbers generally declined in both groups from week 20 onwards. Following work reported by Birk et al., which showed that human infants vaccinated on different days post-birth possessed different numbers of regulatory T cells, with the highest numbers identified in infants vaccinated one day after birth, we applied a similar analysis to the non-human primate data to determine whether the interval between birth and BCG vaccination influenced the number of regulatory T cells measured in the blood. We observed the highest number of regulatory T cells were measured in animals vaccinated 2 days after birth (Supplementary data), although the association was not statistically significant.

Initially at week six post-vaccination there was a trend for lower numbers of γδ T-cells in the BCG vaccinated group (*p* = 0.4560) (Fig. 3D), although subsequently both vaccinated and unvaccinated groups showed similar trends for numbers to increase up to week 36 and then decrease to week 88. The number of NK T-cells was very similar between groups and fluctuated during the study (Fig. 3E). NK cells were initially significantly higher in the vaccinated group (*p* = 0.0455), after which numbers increased steadily to week 36 in both groups (Fig. 3F). Between weeks 36 and 52, NK cell counts fell in both groups, after which, the groups diverged such that numbers decreased in the vaccinated animals and increased in the unvaccinated.

Three populations of monocytes were identified using the markers CD14 and CD16: classical (CD14+ CD16−), non-classical (CD14− CD16+) and intermediate (CD14+ CD16+). The numbers of non-classical monocytes were similar between groups and fluctuated over the time course (Fig. 3G). The level of intermediate monocytes remained relatively stable in both groups for the first 20 weeks of life and then decreased (Fig. 3H). The classical monocytes initially showed a strong trend to be higher in the BCG group (*p* = 0.0597), and both groups showed a steady increase in numbers from weeks 36 to 88 (Fig. 3I).

### BCG vaccination causes early but short-lived increase in PPD-specific IFNγ production

**Figure 4:**
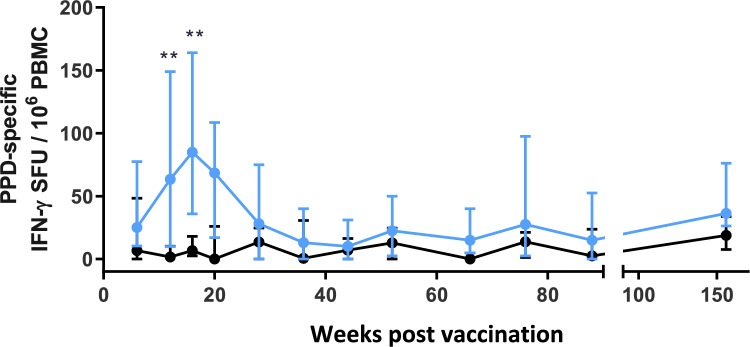
The frequency of PPD-specific secreting IFNγ cells in peripheral blood collected measured using an ex-vivo ELISPOT assay during the first 18 months of life. Blue: BCG vaccinated (n = 34), black: unvaccinated (n = 9). Medians shown with interquartile range. Mann–Whitney-U tests between groups carried out where *p   ≤ 0.05, **p   ≤ 0.01. Data for weeks 6–20 were previously published as part of a larger data set by Sarfas et al.2021^13^.

PBMCs were stimulated with tuberculin PPD and antigen-specific IFNγ producing cells were measured using an IFNγ ELISPOT assay over a 3-year period (Fig. 4). During the initial 16-weeks after vaccination, significantly higher numbers of IFNγ spot forming units (SFU) were detected in the BCG vaccinated animals (week 12: *p*   ≤ 0.0011; week 16: *p*   ≤ 0.0072) then from week 36 onwards, the responses were equivalent in both groups.

### Aerosol boost with BCG, distal to infant BCG vaccination led to increased PPD-specific IFNγ production and changes in cell populations in the BAL and PBMC compartments

**Figure 5:**
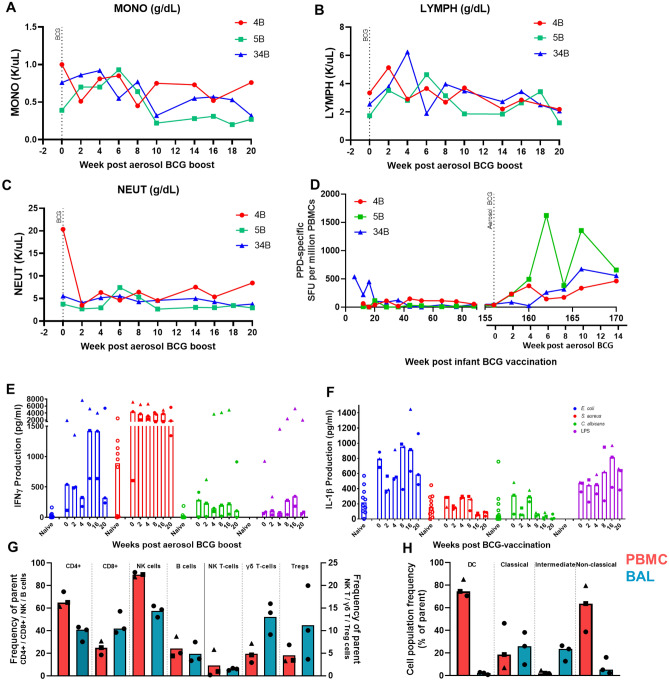
Monitoring immune parameters following aerosol BCG revaccination, three years after initial infant BCG vaccination. (**A**) Concentration of monocyte, (**B**) lymphocyte, and (**C**) neutrophil populations in peripheral blood measured using a haematology analyser, median shown and 95% CI, (**D**) Frequency of PPD-specific IFNγ secreting cells measured by ELISPOT in peripheral blood after initial BCG vaccination in infancy and following aerosol BCG revaccination approximately 3 years later. Individual animals shown (**E**) IFNγ production (pg/ml) following stimulation of PBMC with *E. coli* (blue), *S. aureus* (red), *C. albicans* (green) or LPS (purple).Responses in separate naïve adult animals are shown as a comparison (hollow circles), (**F**) IL1β production (pg/ml) following stimulation of PBMC with *E. coli* (blue), *S. aureus* (red), *C. albicans* (green) or LPS (purple). Responses in separate naïve adult animals are shown as a comparison (hollow circles), (**G**) immunophenotyping of lymphocyte populations in the PBMC and BAL. Samples are gated as a proportion of parent population (CD4+, CD8+, NK T cells, γδ T cells from CD3+ population, B cells from lymphocytes, NK cells from CD3− CD8+ cells, and Tregs from CD3+ CD4+ cells), (**H**) immunophenotyping of monocyte populations in PBMC and BAL Samples are gated as a proportion of parent population (DCs from CD3− CD14− CD8− CD20− CD159− HLA-DR+ population, monocytes from total monocytes). Refer to supplementary data for full gating strategies. Red:  PBMC, blue:  BAL. Bars indicate median values with individual values shown by symbols.

Three animals neonatally primed with BCG were revaccinated using BCG delivered by the aerosol route at approximately 3 years old. Two weeks after aerosol revaccination the number of neutrophils measured in peripheral blood decreased, whereas lymphocyte counts increased (Fig. 5B, C). Lymphocyte numbers had returned to pre-revaccination levels four weeks later, and then remained stable for the rest of the time course. Neutrophil numbers also returned to pre-revaccination levels 4 weeks after aerosol BCG delivery but declined further from week 14 onwards (Fig. 5C). The number of circulating monocytes increased between weeks 2 to 6 but decreased at week 10 following the aerosol BCG revaccination and continued to decline to week 20 (Fig. 5A). Frequencies of PPD-specific IFNγ SFU in peripheral blood greater than those measured prior to revaccination were detected in all animals using an ex-vivo ELISPOT assay, with revaccination-induced responses evident from 2 weeks onward in two of the animals, and from 6 weeks after aerosol BCG revaccination in the third animal (Fig. 5D). The frequencies of PPD-specific IFNγ SFU remained at a higher level after revaccination until the end of the study, in comparison to the 20-week period after the initial BCG vaccination at birth.

Induction of trained immunity was assessed by the measurement of the cytokines IFNγ and IL1β secreted by PBMCs in response to stimulation with microorganisms unrelated to TB, such as *Escherichia coli* (*E. coli*), *Staphylococcus aureus* (*S. aureus*), *Candida albicans *(*C. albicans*) and lipopolysaccharide (LPS). PBMCs collected prior to aerosol BCG revaccination secreted IFNγ in response to *E. coli* and *S. aureus* stimulation, with levels of IFNγ higher after *S. aureus* stimulation (Fig. 5E). At week 8 and 16 after aerosol BCG revaccination, the median IFNγ levels secreted in response to *E. coli* simulation increased, whereas *S. aureus* and *C. albicans* stimulation induced titres remained stable. IL1β secretion was highest at weeks eight and 16 following stimulation with *E. coli* (Fig. 5F). Levels of IL1β decreased two weeks after aerosol BCG and increased at week eight following stimulation with *E. coli*, *C. albicans* and *S. aureus*.

At the end of the study, 20 weeks after delivery of the aerosol BCG revaccination, immunophenotyping was performed on PBMCs and cells collected by bronchoalveolar (BALC) just prior to necropsy. The results revealed differences in CD4:CD8 ratios measured in PBMC and BAL samples, with a higher proportion of CD4+ T-cells measured in PBMCs, in comparison to an equal proportion of CD4+ and CD8+ T-cells detected in the BAL (Fig. 5G). Furthermore, a greater proportion of lymphocytes measured in the BAL were γδ T-cells and Tregs in comparison to PBMCs, although NK cell frequencies were higher in PBMCs and levels of B-cells and NK T-cells were similar in the BALC and PBMC samples. In terms of monocyte derived cells, a higher proportion of dendritic cells (DC) and non-classical monocytes (CD14− CD16+) were determined in PBMC samples, whereas a higher proportion of intermediate monocytes (CD14+ CD16+) were detected in the BAL (Fig. 5H).

## Discussion

In this study, we aimed to monitor the early changes in the cellular immune compartment in infant rhesus macaques as they matured and observe the effect of BCG vaccination on a range of immune cell subsets. Overall, BCG administered shortly after birth did not cause any long-term changes in the number of granulocyte, monocyte, or lymphocyte populations which suggests that BCG did not affect the composition or development of the immune system. However, the data does indicate that cell population counts fluctuate over the first two years of life, which could have an influence on the outcome of immunisations given at different ages. It has been shown previously, that delaying BCG vaccination to 10 weeks after birth, instead of near to birth, generated higher levels of cytokine producing CD4+ T-cells^11^, which could be due to fluctuations in cell populations and developmental changes in the immune compartment, as can be seen in Treg, NK cell, CD14+ CD16+ and CD14− CD16+ monocyte populations between the age of 6 and 12 weeks old as we demonstrate in our data.

Comparison of infant and adult cell populations, revealed CD4+ T-cells, Tregs and NK cell numbers were higher in infants than in adults, a similar difference in these cell populations has also been observed between human infants and adults^12^.This suggests that the development of the cellular immune compartment in NHPs is broadly similar to that in humans, and that the neonatal immunisation model is a relevant platform for studying infant vaccination regimens. NK cells are an important component of the innate immune system and the increased NK cell numbers determined in very young macaques may highlight a reliance on the innate immune response in infants. Tregs are immunomodulatory and moderate pro-inflammatory immune responses that could be potentially harmful to the host if left unchecked. Infant immune responses are also thought to be skewed towards a Th2 profile immune response, which may also guard against inappropriate and damaging immune responses to the new stimuli they are bombarded with^23^. Treg populations have also been shown to increase after BCG vaccination in human infants^10^ and may be responsible for dampening down the pro-inflammatory response, resulting in the lower levels of BCG-induced IFNγ production reported in human and macaque infants^13^.

Recent studies suggest that BCG causes epigenetic changes to monocytes and NK cells^24,25^, leading to trained innate immunity which may be responsible for the heterologous protective effects conferred by BCG against non-mycobacterial pathogens. Although we did not measure epigenetic changes directly in this study because of the limited number of samples, an increase in CD14+ CD16+ and CD14− CD16+ monocytes were observed 6 weeks after infant BCG vaccination, which indicates cell populations implicated in trained immunity were influenced by the BCG vaccination and that training of monocyte cell populations may have occured.

Following aerosol BCG revaccination, three years after the initial infant BCG immunisation, a higher frequency of PPD-specific IFNγ SFU was measured in the peripheral blood of all three macaques compared to the frequencies measured in the equivalent period after neonatal BCG immunisation. We did not see the expected rapid increase after aerosol revaccination, that has been seen in previous studies^20^ suggesting that the long interval between primary vaccination and revaccination did not result in a typical anamnestic response. Macaque 34B made the earliest and highest response to BCG given shortly after birth, but the slowest response to the booster vaccination, whereas macaque 5B made the lowest response to the first BCG vaccination, but highest response to the aerosol boost, which may suggest a blocking or masking effect of the primary vaccination, although the small number of animals in this pilot study means further work would be required to confirm this observation.

Cytokine secretion in response to stimulation with *E. coli, S. aureus* and *C*. *albicans* was used as an indirect measure of trained immunity. The concentration of IFNγ and IL1β detected in response to stimulation with each of the non-mycobacterial antigens before animals received BCG revaccination were higher in PBMC collected from the macaques neonatally vaccinated with BCG, than in those collected from unvaccinated adult macaques, suggesting that heterologous immune responses were detectable up to three years after infant BCG vaccination.

Immunophenotyping of PBMCs and BAL collected at the end of the study showed differences in the frequencies of CD4+ and CD8+ T-cells, NK cells, DC cells, Tregs, γδ T-cells and non-classical monocytes between the systemic (blood) and mucosal (lung) compartments, which likely reflects the different environments and stresses in these immune compartments. There were higher proportions of Tregs in the BAL, probably due to the requirement to dampen down potentially inappropriate responses induced by exposure to an increased level of environmental stimuli. Higher proportions of γδ T-cells were also found in the BAL, which is expected as their role in the first line of defence, is to be ready to rapidly respond and produce cytokine, and they are often enriched in mucosal surfaces. The proportions of lymphocyte cell populations measured in the blood after aerosol BCG revaccination were broadly similar to those determined in unvaccinated adult macaques, suggesting that revaccination did not affect these populations. In contrast, monocyte populations changed from the more typical bias toward a classical phenotype in the unvaccinated cohort^13,26^, to a phenotype biased towards a non-classical immunomodulatory phenotype in the aerosol BCG revaccinated cohort.

The regular sampling of individuals undertaken in this study has revealed natural fluctuations in cell populations, that could have been missed in single time point analysis of samples collected from groups of differing individuals. Further work is required to better understand the long-term changes in the cellular immune compartment following vaccination of infants, and whether the memory laid down in infancy has a protective effect later on, and how durable this protection may be in NHPs. This study has demonstrated similarities between BCG vaccinated infant macaques and humans and provides evidence that infant vaccinated NHPs are a relevant model for evaluation of vaccines targeting infant populations and vaccines designed to boost infant BCG vaccination induced immunity.

## Materials and methods

### Experimental animals

Forty-three male and female rhesus macaques (*Macaca mulatta*) of Indian genotype were sourced from an established, characterised, closed UK breeding colony. Compatible social groups were housed in accordance with Home Office (UK) and NC3Rs guidelines in cages with high-level observation balconies, extensive environmental stimulation and provided with a wide range of dietary enrichment. Animals were monitored throughout the study for changes in weight, temperature, haemoglobin levels and changes in behaviour. Animal procedures and study designs were approved by the UKHSA Establishment Animal Welfare and Ethical Review Committee, Porton Down UK and authorised under a UK Home Office project licence. The manuscript has been prepared in compliance with the ARRIVE guidelines.

### Study design—infant vaccination

34 macaques were BCG vaccinated within seven days of birth and nine age-matched controls were sampled alongside. Animals were vaccinated with an infant’s dose (50 μl, approx. 3.65 × 10^5^ CFU) of BCG Danish 1331 (SSI/AJ Vaccines) prepared according to manufacturer’s instructions and delivered intradermally into the upper left arm. Blood samples were collected at regular intervals over the following 88 weeks and subsequently at week 152. Blood samples were collected from a further group of 12 naive adult rhesus macaques (~ 3 years old) and used to compare the frequency and characteristics of cell populations in infants and adults.

### Study design—aerosol boost with BCG at ~ 3 years old

Three animals from the original cohort of BCG vaccinated infants were re-vaccinated three years after their primary vaccination with BCG in infancy with BCG delivered at a dose of 1 × 10^7^ CFU by the aerosol route using the Omron nebuliser as described elsewhere^19^. Blood samples were collected at two weekly intervals for 20 weeks following aerosol BCG. BAL samples were collected at the end of the study using a bronchoscope as described previously^27^.

### IDEXX ProCyte DX

Blood samples anti-coagulated with EDTA or heparin, (both BD Biosciences, USA). All ProCyte DX were analysed using an IDEXX ProCyte DX Haematology analyser (IDEXX, USA). Blood from infants under 20 weeks old was diluted 1:4 with R0 media (comprised of RPMI supplemented with L-glutamine, penicillin/streptomycin solution, 2-mercaptoethanol and Hepes buffer (all Sigma-Aldrich, UK) due to the limited volumes of blood permitted for collection from the animals. Results are expressed as absolute counts (K/µl), or ratios thereof.

### Whole blood immunophenotyping

Whole Blood Immunophenotyping assays were performed using 50 µl of heparinised blood incubated for 30 min at room temperature with optimal dilutions of the following antibodies: anti-CD3-AF700, anti-CD4-APC-H7, anti-CD8-PerCP-Cy5.5, anti-CD95-Pe-Cy7, anti-CD14-PE, anti-HLA-DR-BUV395, anti-CD25-FITC (all from BD Biosciences, UK); anti-CD127-APC (eBioscience, Hatfield UK); anti-γδ-TCR-BV421, anti-CD16-BV786, anti-PD-1-BV711, anti-CD20-PE-Dazzle (all from BioLegend, London UK); and amine reactive fixable viability stain red (Life Technologies, Paisley, UK); all prepared in brilliant stain buffer (BD Biosciences, UK). Red blood cell contamination was removed using a Cal-lyse reagent kit as per the manufacturer’s instructions (Thermofisher Scientific, UK). BD Compbeads (BD Biosciences, UK) were labelled with the above fluorochromes for use as compensation controls. Following antibody labelling, cells and beads were fixed in a final concentration of 4% paraformaldehyde solution (Sigma Aldrich, UK) prior to flow cytometric acquisition.

Cells were analysed using a five laser LSRII Fortessa instrument (BD Biosciences, UK) and data were analysed using FlowJo (version 10, Treestar, Ashland, USA). Immediately prior to flow cytometric acquisition, 50 µl of Truecount bead solution (Beckman Coulter, USA) was added to each sample. Leukocyte populations were identified using a forward scatter-height (FSC-H) versus side scatter-area (SSC-A) dot plot to identify the lymphocyte, monocyte and granulocyte populations, to which appropriate gating strategies were applied to exclude doublet events and non-viable cells. Lymphocyte sub populations including T-cells, NK-cells, NKT-cells and B-cells were delineated by the expression pattern of CD3, CD20, CD95, CD4, CD8, CD127, CD25, CD16 and the activation and inhibitory markers HLA-DR and PD-1. GraphPad Prism (version 8.0.1) (GraphPad Software Inc, USA) was used to generate graphical representations of flow cytometry data. Gating strategy can be found in the Supplementary Data.

### IFNγ ELISPOT

ELISPOT assays were performed on peripheral blood mononuclear cells (PBMC) isolated from heparin anti-coagulated blood using standard procedures, to measure the frequency of cells producing Interferon-gamma (IFNγ) in response to stimulation with 10 µg/ml purified protein derivative (PPD) (SSI, Copenhagen, Denmark) using MabTech ELISPOT kits (Mabtech, Sweden), as previously described^20^.

### PBMC and BAL immunophenotyping

At necropsy, PBMCs and cells collected by BAL were isolated using standard methods. 1 × 10^6^ cells were used for immunophenotyping using the panel described for whole blood immunophenotyping. After staining for 30 min at room temperature, samples were centrifuged and resuspended in 4% paraformaldehyde and analysed using a LSRII Fortessa flow cytometer (BD Biosciences, UK) as described above.

### Trained immunity assay

Cryopreserved PBMCs were thawed and resuspended in warm R10 media and 1 U/ml DNase (Sigma Aldrich, UK), followed by centrifugation at 400 rpm. This process was repeated twice. R10 media was composed of RPMI 1640 supplemented with 2 mM l-glutamine, 50 U/ml penicillin/50 µg/ml streptomycin, 25 mM HEPES buffer, 55 mM 2-mecaptoethanol (all Sigma Aldrich, UK) and 10% heat-inactivated, filtered foetal calf serum (Labtech Ltd., Uckfield, UK). Following the second centrifugation, PBMCs were resuspended in R10, a viable cell count was performed, and cells were adjusted to reach a final concentration of 5 × 10^5^ cells/200 µl.

*Escherichia coli, Staphylococcus aureus* and *Candida albicans* were obtained from C. Hind from UKHSA and cultured, quantified, heat killed, aliquoted and stored at −80 °C. Aliquots of the heat-killed *E. coli, S. aureus* and *C. albicans* were defrosted and diluted in phosphate buffer solution, to a final concentration of 1 × 10^6^ cells/ml and the lipopolysaccharide (LPS) (Sigma Aldrich, UK) was diluted in deionised water to a final concentration of 1 ng/ml, alongside a PBS negative control stimulation. To a 96-well plate, the stimulants were pipetted into separate wells and 5 × 10^5^ cells added. The plate was incubated for 24 h at 37 °C and 5% CO_2_, following which supernatants were harvested and stored at -80 °C.

### IFNγ and IL-1β ELISA

IFNγ and IL-1β cytokine levels in the supernatants from the stimulated PBMC cultures were measured using an IFNγ ELISA kit (MabTech, Stockholm, Sweden) and IL-1β ELISA kit (R&D Systems, Minneapolis, MN, USA) according to the manufacturer’s instructions. The concentrations of cytokine were calculated using the standard curves from the kit, and the PBS negative controls were subtracted from the microorganism-specific wells. Data was analysed using GraphPad Prism (version 8.0.1).

### Statistical analysis

Whole blood immunophenotyping data was analysed using Microsoft Excel, and absolute counts calculated using the TrueCount beads by the calculation: (number of cell events/number of bead events) x (assigned bead count of the lot). IFNγ ELISPOT and trained immunity data was analysed in Microsoft Excel to background subtract the unstimulated samples from the antigen stimulated samples. All data was subject to further analysis using GraphPad Prism (version 8.0.1) and subject to statistical tests including Mann–Whitney U tests.

### Ethics statement

Animal procedures and study designs were approved by the UKHSA Establishment Animal Welfare and Ethical Review Committee, Porton Down UK and authorised under a UK Home Office project licence. The manuscript has been prepared in compliance with the ARRIVE guidelines.

### Supplementary Information

Supplementary Figures.

## Data Availability

All data generated or analysed during this study are included in this published article (and its Supplementary Information files).
