# Supplementary material for: Immune cell population dynamics following neonatal BCG vaccination and aerosol BCG revaccination in rhesus macaques
Source: Sci Rep. 2024 Jul 23;14:16993. doi: 10.1038/s41598-024-67861-6 (PMC11266652; doi:10.1038/s41598-024-67861-6)
Supplement: Supplementary file 1 — Supplementary Figures. [file 41598_2024_67861_MOESM1_ESM.docx]

**Immune cell population dynamics following neonatal BCG vaccination and aerosol BCG revaccination in rhesus macaques.**

Laura Sibley^1^, Charlotte Sarfas^1^, Alexandra Morrison^1^, Jessica Williams^1^, Konstantinos Gkolfinos^1^, Adam Mabbutt^1^, William Eckworth^1^, Steve Lawrence^1^, Mike Dennis^1^, Andrew White^1^, Sally Sharpe^1^.

^1^UK Health Security Agency, Porton Down, Salisbury SP4 0JG

**Supplementary data**

**
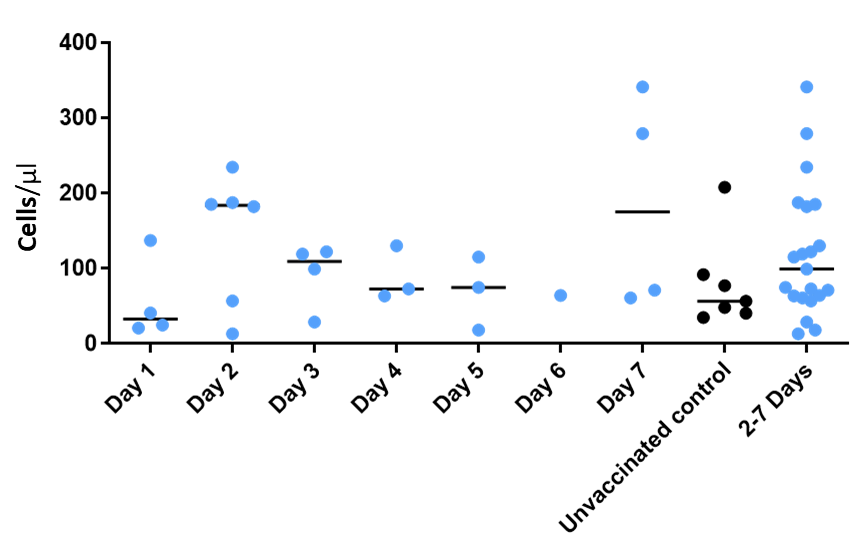
**

Supplementary Figure 1. Treg numbers at week 12 post-BCG vaccination depending on which day after birth they were vaccinated on. Medians shown and dots indicate individuals.


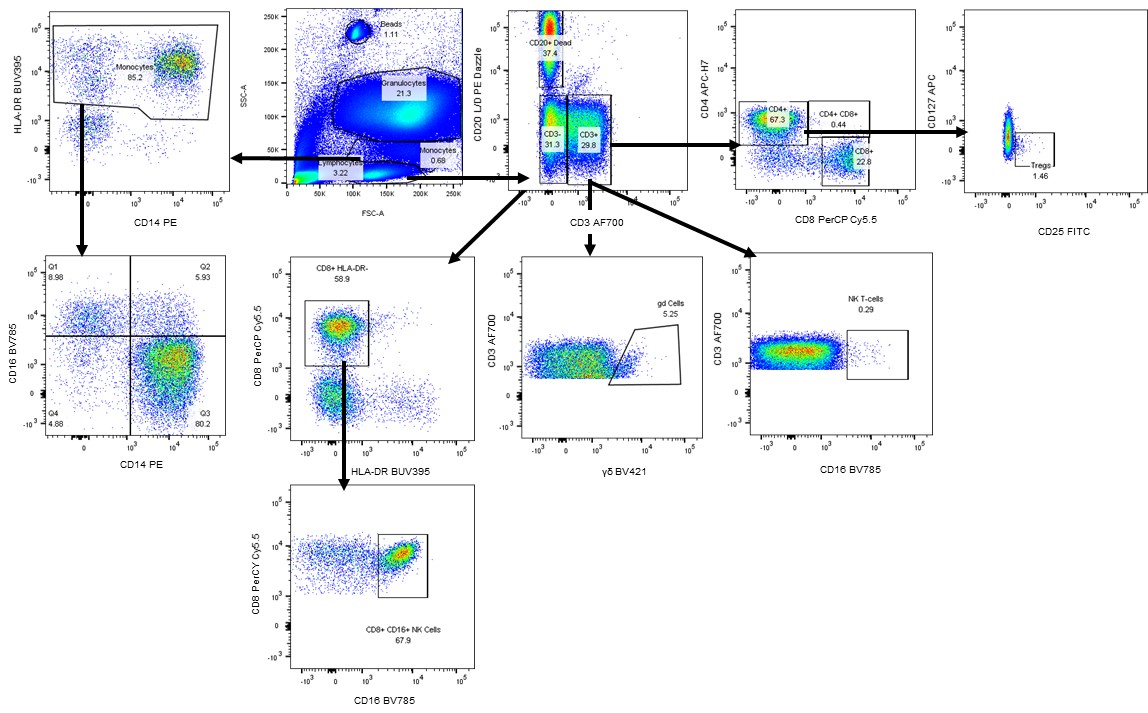


Supplementary Figure 2. Gating strategy for flow cytometry panel of whole blood, PBMC and BAL immunophenotyping.


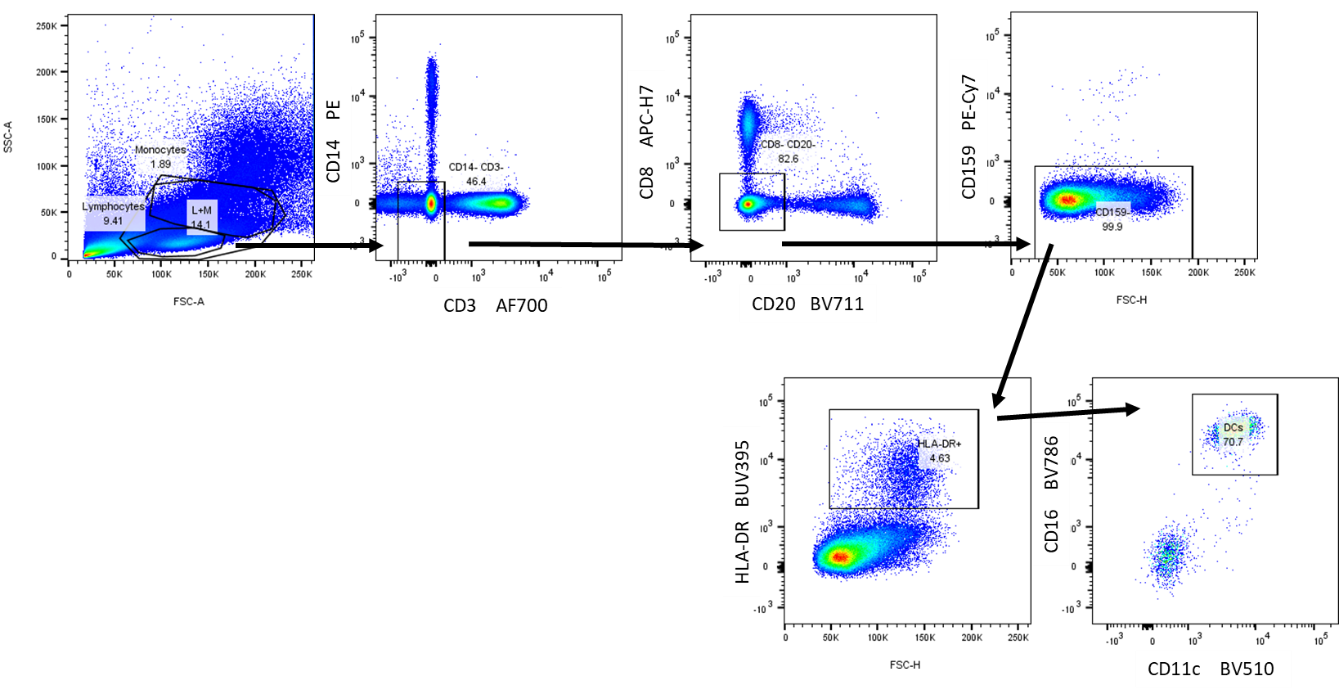


Supplementary Figure 3. Gating strategy for flow cytometry panel of PBMC and BAL to determine dendritic cell (DC) populations.
